# Receptor-like cytoplasmic kinases VII subfamily orchestrates reactive oxygen species signaling in plant immunity

**DOI:** 10.1080/15592324.2026.2685951

**Published:** 2026-06-10

**Authors:** Ping Li, Yan Liang

**Affiliations:** a State Key Laboratory for Quality and Safety of Agro-Products, Key Laboratory of Biotechnology in Plant Protection of MARA, Zhejiang Key Laboratory of Green Plant Protection, Institute of Plant Virology, Ningbo University, Ningbo, China; b Department of Plant Protection, Institute of Biotechnology, Zhejiang University, Hangzhou, China

**Keywords:** Receptor-like cytoplasmic kinase, reactive oxygen species, respiratory burst oxidase homolog, NADPH oxidase, plant immunity

## Abstract

Receptor-like cytoplasmic kinases (RLCKs) are a large family of receptor-associated kinases that lack extracellular and transmembrane domains, yet they function as key signaling components downstream of receptor-like kinases. In recent years, accumulating evidence has established members of the RLCK VII subfamily as central regulators of plant immune signaling, particularly in controlling reactive oxygen species (ROS) production during immune activation. Here, we summarize recent advances in understanding how RLCK VII subfamily members in Arabidopsis orchestrate ROS signaling.

Reactive oxygen species (ROS) production represents one of the earliest and most conserved responses during plant immune activation. Beyond their direct antimicrobial effects, ROS also function as key signaling molecules that amplify downstream defense responses.^1^ In *Arabidopsis*, ROS signaling is primarily mediated by respiratory burst oxidase homolog D (RBOHD), a plasma membrane-localized NADPH oxidase. RBOHD activity is finely tuned by multiple posttranslational modifications, such as phosphorylation,^2-6^ nitrosylation,^7^ ubiquitination,^5^ and phosphatidic acid binding.^8-10^ Among these, Receptor-like cytoplasmic kinases (RLCK)-mediated phospho-relay represents a major mechanism for RBOHD activation. Over the past three decades, extensive studies have focused on RLCKs, particularly those in the RLCK VII subfamily, also known as the PBS1-like (PBL) family.^11^ ^,^ ^12^ In *Arabidopsis*, the RLCK VII subfamily contains 46 members that can be further divided into nine groups, named RLCK VII-1 to RLCK VII-9.^11^ ^,^ ^13^ ^,^ ^14^ With respect to ROS signaling, members of this subfamily generally function redundantly; however, specific groups exhibit distinct regulatory roles depending on the elicitor (Figure 1).^4^ ^,^ ^11^ ^,^ ^15-19^

Botryis-induced kinase 1 (BIK1), a member of the VII-8 group, was the first RLCK reported to regulate ROS production upon perception of flg22, a conserved epitope of flagellin.^20^ Accordingly, the T-DNA insertion mutant *bik1-1* exhibits reduced ROS levels upon flg22 treatment. Subsequent studies revealed that the *bik1-1* mutant also shows impaired ROS production when treated with other immune elicitors, including Pep1 (plant elicitor peptide 1), chitin, and elf18 (a conserved fragment of bacterial elongation factor Tu).^2^ ^,^ ^3^ ^,^ ^21^ However, it displays increased ROS production upon treatment with nlp20, a conserved 20-amino-acid fragment found in most necrosis and ethylene-inducing peptide 1-like proteins.^22^ Recent work using CRISPR-Cas9-generated *bik1* mutants largely recapitulates the responses of *bik1-1* to most elicitors, but, notably, exhibits reduced (rather than increased) nlp20-induced ROS production. This discrepancy has been attributed to potential genomic perturbations in *bik1-1* mutant lines.^23^ Moreover, PBL1, another member of this subgroup also regulates the flg22-, elf18-, nlp20- and Pep1- triggered ROS burst, and *bik1 pbl1* double mutants show a stronger reduction in ROS production compared with the corresponding single mutants.^23^

Beyond the VII-8 group, the role of the VII-6 group in regulating ROS production has also been well studied, particularly in the case of RPM1-induced protein kinase (RIPK).^4^ ^,^ ^24^ It has been shown that *ripk* mutants generated by T-DNA insertion, RNAi interference, point mutation, or CRISPR-Cas9 editing all consistently exhibit reduced ROS production in response to the above-mentioned elicitors.^4^ Moreover, RIPK is also essential for ROS production triggered by the bacterial medium-chain 3-hydroxy fatty acid metabolite 3-OH-C10:0, various bacterial effectors, pipecolic acid, and methyl salicylate.^4^ Notably, RIPK and BIK1 function redundantly in flg22- and elf26- triggered ROS production, as *bik1-1 ripk-3* double mutants display a stronger reduction in ROS accumulation than either single mutant.^4^ RIPK and BIK1 also share similar regulatory mechanisms in controlling ROS production, including direct phosphorylation of RBOHD^2-4^ as well as indirect regulation via phosphatidic acid production to stabilize RBOHD and sustain ROS generation.^9^ ^,^ ^10^ In addition, RIPK sustains ROS production, in part, by activating NADP-malic enzyme 2, thereby increasing the NADPH pool to fuel RBOHD.^25^ Together, these studies demonstrate that members of the RLCK VII subfamily, particularly BIK1 and RIPK, are essential positive regulators of broad-spectrum ROS production during plant immune responses.

In contrast, several other members within the VII-6 group act as negative regulators of ROS production (Figure 1). PBL13, for instance, has been shown to suppress RBOHD protein abundance, and its mutation results in an enhanced ROS level upon flg22 or chitin treatment. Similarly, a *pbl8 pbl16 pbl17* triple mutant also displays elevated ROS accumulation under the same conditions.^10^ ^,^ ^11^ ^,^ ^16^ In addition, mutation of *Constitutive Differential Growth 1* (*CDG1*), another member within the RLCK VII family, enhances ROS production in response to flg22.^26^ Such functional diversification within the RLCK VII family underscores the complexity of RLCK-mediated signaling networks during plant immune responses.

Collectively, these studies highlight the central role of RLCKs in coordinating RBOHD-mediated ROS generation. Despite this substantial progress, many aspects of RLCK-mediated ROS signaling, particularly its functions in crop species, remain poorly understood. (1) *Role of RLCK VII in abiotic stress*. ROS signaling also plays important roles in abiotic stress, such as drought and salinity. It therefore warrants investigation whether the RLCK VII subfamily orchestrates ROS production in response to such stress. (2) *Evolution of functional diversification within the RLCK VII subfamily*. RIPK and PBL13 are closely related homologs within the RLCK VII subfamily, yet they exert opposing effects on ROS production. It would be interesting to understand how this functional divergence arose during evolution, whether through sequence variation or structural changes. (3) *Structural basis of RLCKs*. High-resolution structural information for full-length RLCKs is lacking, making it difficult to understand how conformational changes, autophosphorylation, or interactions with receptors modulate their activities and determine whether individual RLCKs adopt specialized or broader molecular functions in plant immunity. A deeper understanding of these questions may ultimately facilitate the rational engineering of disease-resistant crops with minimal fitness costs.

**Figure 1.**
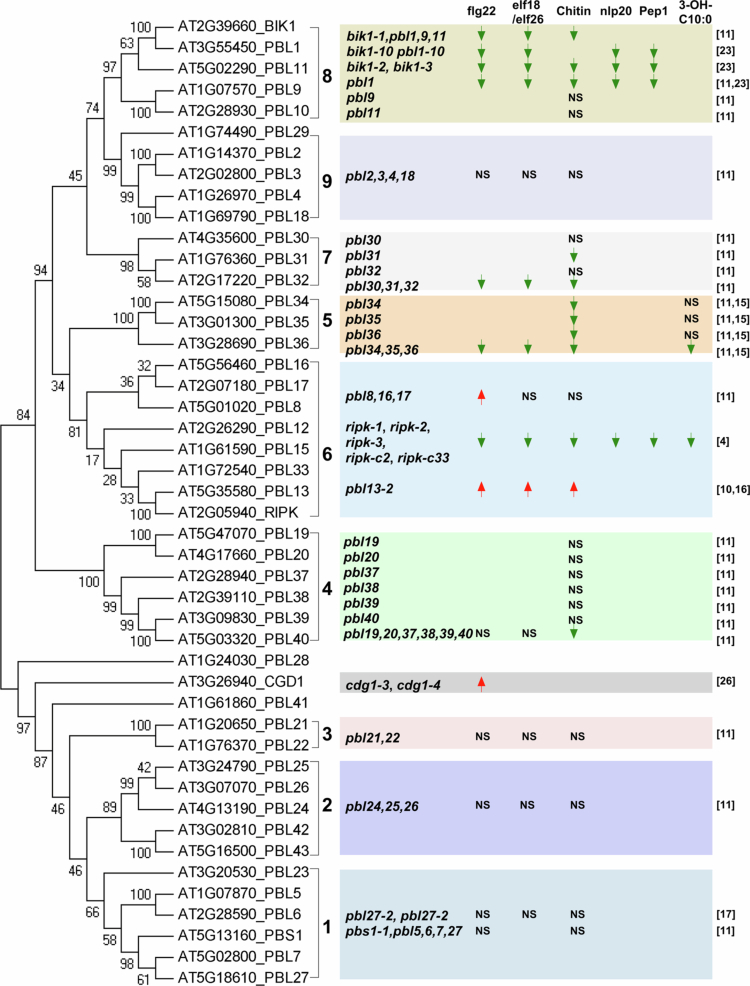
Function of RLCK VII family members in elicitor-induced ROS production. The phylogenetic tree was constructed using the kinase domains of the RLCKs with the neighbor-joining method and 1000 bootstrap replicates in MEGA 5. Subgroup classification follows Hailemariam et al.^13^ The horizontal axis lists six elicitors: flg22, elf18/elf26, chitin, nlp20, pep1, and 3-OH-C10:0. Red upward arrows indicate increased ROS accumulation, green downward arrows indicate reduced ROS accumulation, and “NS” indicates no significant difference.
